# Statistical Assessment of Drug Synergy from *In Vivo* Combination Studies Using Mouse Tumor Models

**DOI:** 10.1158/2767-9764.CRC-23-0243

**Published:** 2023-10-23

**Authors:** Binchen Mao, Sheng Guo

**Affiliations:** 1 Crown Bioscience Inc., Suzhou, Jiangsu, P.R. China.

## Abstract

Drug combination therapy is a promising strategy for treating cancer; however, its efficacy and synergy require rigorous evaluation in preclinical studies before going to clinical trials. Existing methods have limited power to detect synergy in animal studies. Here, we introduce a novel approach to assess *in vivo* drug synergy with high sensitivity and low false discovery rate. It can accurately estimate combination index and synergy score under the Bliss independence model and the highest single agent (HSA) model without any assumption on tumor growth kinetics, study duration, data completeness and balance for tumor volume measurement. We show that our method can effectively validate *in vitro* drug synergy discovered from cell line assays in *in vivo* xenograft experiments, and can help to elucidate the mechanism of action for immune checkpoint inhibitors in syngeneic mouse models by combining an anti-PD-1 antibody and several tumor-infiltrating leukocytes depletion treatments. We provide a unified view of *in vitro* and *in vivo* synergy by presenting a parallelism between the fixed-dose *in vitro* and the 4-group *in vivo* combination studies, so they can be better designed, analyzed, and compared. We emphasize that combination index, when defined here via relative survival of tumor cells, is both dose and time dependent, and give guidelines on designing informative *in vivo* combination studies. We explain how to interpret and apply Bliss and HSA synergies. Finally, we provide an open-source software package named invivoSyn that enables automated analysis of *in vivo* synergy using our method and several other existing methods.

**Significance:**

This work presents a general solution to reliably determine *in vivo* drug synergy in single-dose 4-group animal combination studies.

## Introduction

Combination therapies using two or more therapeutic agents can be superior to single-agent therapies in the treatment of cancers (1–3). They can be more efficacious and overcome treatment resistance by targeting different proteins, pathways, and cell populations, or reduce toxicity by using lower dose of constituent agents, or broaden the cancer types for treatment. However, the combination of two therapeutic agents or drugs may not be effective or even counterproductive, because the combined effect can be additive, synergistic, or antagonistic if it is equal to, greater, or weaker than the sum of the two drugs’ individual effects. Only synergistic effect or synergy is desired.

Combination therapies are first evaluated in preclinical *in vitro* and *in vivo* models before going to clinical trials. High-throughput screenings in cancer cell lines have produced promising hits (4–6), and experimental approaches remain the definitive method despite recent progress in the computational prediction of drug combination effects (7). Methods for assessing *in vitro* combination effects are well established with many software packages (8–13), which implement major synergy models including Loewe additivity (14), Bliss independence (15), highest single agent (HSA; ref. 16), and zero interaction potency (ZIP; ref. 17), and more recently the unifying MuSyC synergy framework that mechanistically distinguishes and statistically separates potency synergy and efficacy synergy (13, 18). The models differ in assumptions on single-agent pharmacology and shall be used and interpreted with a good understanding of drug mechanisms (19). There are a variety of study designs including single concentration (only one concentration for each drug), anchored concentration (fixed concentration for one drug), fixed concentration ratio, matrix or full factorial design, ray design, and cross designs (19–21). For all designs, direct calculation, or dose–response curves, or surface methods are used to determine proper drug concentration and to fit synergy models with statistical evaluation.

*In vitro* synergy is often further validated in mouse tumor models such as patient-derived xenografts (PDX), cell line–derived xenografts, and mouse syngeneic models (4–6, 22). A typical *in vivo* combination study consists of four treatment groups for the vehicle control, drug A, drug B, and drug A+B, with fixed dose for each drug. A group has multiple mice as biological replicates for estimating variance and improving statistical power. Tumor volume (TV) is measured every few days to describe the longitudinal tumor growth. Different mice may have different numbers of TV datapoints due to early dropout, euthanasia at large TV, extended dosing period under effective treatment, etc. The entirety or part (e.g., on a particular day) of the TV data is used to evaluate the combination effect. Mouse studies are more complex than cell line assays in study design, data acquisition, and analysis. The dose–response curve and surface methods used for cell line assays are no longer applicable because there is only a single dose for a drug (as a result, Loewe and ZIP models are no longer applicable since both require dose–response curves for parameter estimation). It is necessary to use more appropriate methods to accommodate and leverage *in vivo* combination study data.

Research on *in vivo* synergy methodologies has been relatively sparse. Most studies simply compare TV at a single day using *t* test, or use survival analysis to compare the time-to-event (i.e., time for TV to reach a threshold) between groups (4–6, 23). Nonetheless, there has been some effort to establish more rigorous methods. Some representative work is reviewed here. Wu and colleagues applied the Bliss independence model and defined a combination index (CI; called interaction index therein) that is calculated from TV at the last day of a study when there are still TV data in all four groups. They also used linear regression on log-transformed TV at the last day with an explicit regression coefficient to quantify and test the significance of the combination effect (24). Subsequently, Wu defined an alternative Bliss CI based on parameters estimated from survival analysis; however, the index becomes undefined if tumors shrink in any group, which is often seen for efficacious drugs (25). Zhao and colleagues used differential equations to model tumor growth and derived a Bliss independence index (26), which is equivalent to interaction index in Wu and colleagues (24). More recently, Huang and colleagues established CombPDX as a statistical framework for three synergy models: Bliss independence, HSA, and response additive (RA; ref. 27), their definition of Bliss CI is equivalent to that in Wu and colleagues (24), while HSA and RA were newly introduced. TV data at a single day were used to calculate a local CI, the average of all local CI values is the global CI (27). These works were essentially using single-day TV to evaluate synergy. On the other hand, Demidenko and colleagues advanced research on *in vivo* synergy by taking a more global approach, that is, by considering the overall TV dynamics in the study duration (28–30). Specifically, Demidenko and Miller defined Bliss independence using tumor growth rates (GR) of the four groups by assuming exponential tumor growth kinetics, and devised a Z-test to assess whether Bliss synergy exists using estimated GRs obtained from fitting linear mixed models on log-transformed TV data (29).

These methods, however, have inherent limitations because they either impose unrealistic assumptions for tumor growth and TV data, or use only TV data from a single day to calculate CI (local CI values may fluctuate excessively such that they may indicate synergy and antagonism at different days), or have trouble dealing with missing and unbalanced TV data even though mathematical interpolation is used to patch missing data with unmeasurable uncertainty, or that CI cannot be calculated after a day when most or all mice in the vehicle control group are euthanized because their tumors reach certain size threshold faster than drug-treated tumors.

In this study, we developed a robust method for assessing *in vivo* combination effect. Our method estimates a global CI with significance assessment by *P* values and confidence intervals for two synergy models: Bliss independence model and HSA model. We first describe an efficacy readout we previously proposed (31), which, now termed eGR, is based on tumor GR calculated from TV values at multiple timepoints, and is directly comparable with several rate-based metrics for cell assays with longitudinal measurements, including notably the drug-induced GR inhibition (32), and the drug-induced proliferation (DIP) rate (33) that was used in the landmark MuSyC framework (13, 18). We then define CI and synergy score (SS) for Bliss and HSA synergy models following previous works (29, 34). Subsequently, we demonstrate the advantages of our method on two case studies. We used simulation and case studies to show that our method has high sensitivity and low FDR in detecting synergy. Finally, we give a unified view of *in vitro* and *in vivo* synergy study design and interpretation under the Bliss independence model.

## Materials and Methods

### Quantification of Drug Efficacy

**FIGURE 1:**
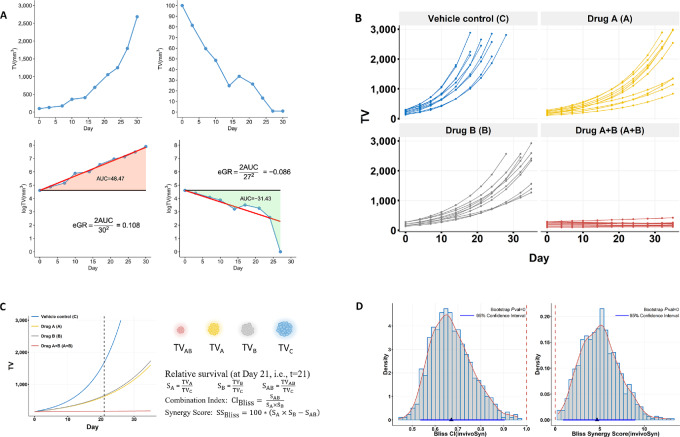
The quantification of *in vivo* synergy based on an unbiased metric of drug effect. **A,** Definition of eGR, an unbiased metric of *in vivo* drug effect. Upper graphs shows two synthetic curves, for illustrative purposes, depicting tumor growth and shrinkage measured by TV. Bottom graphs show the same tumor growth curves in natural log scale, and illustrate the calculation of eGR, which is defined as 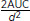, where AUC is the size of the colored area and *d* is the study duration in days (see Materials and Methods for details). **B,** The 4-group design is the most common *in vivo* combination study, which has four treatment groups for the vehicle control, drug A, drug B, and drug A+B, with fixed doses for the two drugs. A group usually has multiple mice that vary in tumor growth curves and number of TV datapoints. **C,** The average tumor growth curves of the four groups. The relative survival is calculated for drugs A, B, A+B based on TV at a particular day, then the CI and SS are estimated under several models, only Bliss independence model is shown. **D,** Bootstrap confidence internals and *P* values are calculated for both CI and SS. The histogram, overlaid by a red fitted density curve, shows the distribution of 1,000 bootstrap values for CI or SS; the red dashed vertical line indicates additive effect (1 for CI and 0 for SS); the black triangle marks the calculated value for CI or SS; the blue horizontal line indicates the 95% confidence interval. invivoSyn is the name of our method as well as the software package implementing it.

We previously defined an efficacy readout called normalized AUC that makes no assumption on tumor growth kinetics and is invariant to starting TV (31). To avoid confusion, we rename it to exponential growth rate or *eGR*, which is the overall tumor GR during the entire study duration under an equivalent exponential kinetics assumption in the sense that if the tumor growth follows an ideal exponential growth equation 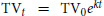, where TV_*t*_ and TV_0_ are TV at the starting day and day *t*, then eGR is just the rate constant *k*; if however, the tumor growth is deviated from the strict exponential kinetics (e.g., a tumor may shrink first then resume growth after developing resistance, or it may keep growing then shrink after the drug takes effect), then eGR is numerically identical to the rate constant *k* of an equivalent exponential growth equation such that the net TV change is the same between the actual tumor growth and this equivalent exponential tumor growth (Fig. 1A).

eGR can be conveniently calculated using simple algebra. Briefly, a tumor growth curve is plotted with the logarithmic scale for TV. The trapezoidal rule is used to calculate the total AUC, which is then subtracted by the area of the rectangle between horizontal lines logTV_0_ and zero to obtain the net-gain AUC, where TV_0_ is the starting TV at day 0 (Fig. 1A). This net-gain AUC is positive, zero, or negative if the overall TV increases, stays the same, or decreases during the whole treatment period from day 0 to day *d*. eGR is then defined as 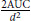, where AUC refers to the net-gain AUC. For a treatment group with multiple mice, we take the mean of all individual eGR values as the eGR for the group. In a mouse combination study, we estimate eGR_C_, eGR_A_, eGR_B_, and eGR_AB_ for treatments with vehicle control, drug A, drug B, and drug A+B.

### Definition of CI and SS

We view TV as a measurement of the number of cells in a tumor. After the estimation of eGR values and their interpretation as rate constants in exponential growth equations, we can use the following formulae to calculate the relative survival (i.e., the surviving fraction of cells) under the three drug treatments (drug A, drug B, drug A+B) with respect to the control treatment (*t* ≥ 0),



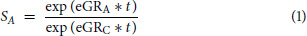





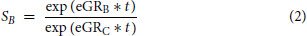





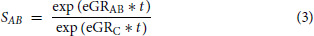



We therefore define the CI as the ratio between observed survival and expected survival.



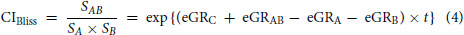





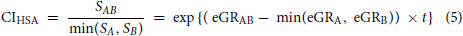



We impose *t* = 21 (3 weeks as the treatment duration) as a convention so that the range of CI conforms to the convention used in cell line studies. Specifically, CI > 1, = 1, <1 indicate antagonism, additivity, and synergy, respectively.

The Bliss SS has been defined as the survival difference in percentage between the expected relative survival and the observed relative survival, as previously described in Demidenko and Miller (29). The HSA synergy follows the definition from Wooten and colleagues (34). SS < 0, = 0, >0 indicate antagonism, additivity, and synergy, respectively.



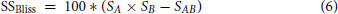





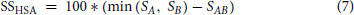



Using the stratified bootstrap method, we can estimate the confidence interval and *P* value for the CI and the SS to evaluate the significance and magnitude of the combination effect between two drugs. Specifically, we randomly draw 1,000 samples (sampling with replacement) from the tumor growth data, with the treatment group serving as the strata, then calculate the bias-corrected and accelerated (BCa) bootstrap interval and *P* value for both CI and SS. BCa bootstrapping is more appropriate for skewed distributions, as is the case here. In particular, both Bliss and HSA combination indices theoretically assume log-normal distributions.

### Simulation Studies and Power Analysis

The exponential tumor growth model (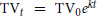) was used to simulate tumor growth data by specifying the initial tumor volume TV_0_ and the rate constant *k*. Treatment effect was modeled by parameter TGI_tr_^21^, which was defined as 1 − RTV_tr_^21^/RTV_c_^21^ to reflect the reduction of relative tumor volume (RTV) at day 21 between the treatment group (Tr) and the control group (C). Parameter T_obs_ was used for modeling the time to follow in this simulation, and parameter TV_max_ was used to represent TV at which the animal was sacrificed and was set to 3,000 mm^3^. Furthermore, parameter T_ki_ was used for modeling treatment kick-in time, a value larger than 0 indicating delayed kick-in. For modeling intrinsic resistance or induced resistance, parameter P_rt_ was used to represent the percentage of tumor cells that is resistant to treatment, and parameter T_ir_ was used to model the day induced resistance occurs. By default, T_ki_, P_rt_, and T_ir_ were all set to 0 to model regular tumor growth curve. Finally, parameters CV_0_ and CV_k_ were used to model coefficient of variance of initial TV and tumor GR among animals, respectively, and parameter σ was used to model the random error of TV measurement.

To simulate the regular tumor growth pattern, we assigned TGI_tr_^21^ values of 0.5 and 0.4 to treatment A and treatment B, respectively. We varied the TGI_tr_^21^ values of the combo treatment A+B from 0.7 to 0.8 for the Bliss model to represent three levels of synergy (nil, weak, and strong).

To simulated the delayed treatment effect, we fixed T_ki_ = 10 days for treatment A and the combo treatment. In addition, we assigned TGI_tr_^21^ values of 0.5, 0.4, and 0.8 to treatments A, B, and A+B, respectively. By varying T_obs_ from 40 to 60 days, we examined the association between power and T_obs_.

To model intrinsic resistance, we assigned a positive P_rt_ = 0.1 to all three drug treatment groups (A, B, A+B). To model induced resistance, we used T_ir_ = 10 days to represent the day when induced resistance occurs at only treatment groups A and A+B. In both cases, treatments A and B were assigned TGI_tr_^21^ values of 0.5 and 0.4, respectively. The combo treatment was assigned TGI_tr_^21^ values of 0.8 and 0.9.

We generated 100 datasets of tumor growth under different parameter combinations and calculated the SSs for the Bliss model. Statistical power was defined as the proportion of datasets with a *P* value less than 0.05 out of the 100 simulations.

### *In Vivo* Combination Effect Evaluation by CombPDX

*In vivo* combination effect was assessed by the CombPDX method at its webserver (https://licaih.shinyapps.io/CombPDX). CombPDX first quantifies treatment effect using the formula δ_g_ = (µ_C_−µ_g_)/µ_C_ for g∈ (A, B, A+B), where µ denotes the mean relative TV at a specific day compared with the start day, subscriptions denote the four groups. For the Bliss model, CI is calculated as log(µ_A_) + log(µ_B_) − log(µ_C_) − log(µ_AB_). CI < 0, CI = 0, and CI > 0 indicate antagonistic, additive and synergistic effects, respectively. The global CI (gCI) is derived by averaging the local CI values at individual days in the study duration.

### *In Vivo* Combination Studies

The *in vivo* combination study using colon PDX model CR1197 was conducted in the specific pathogen-free animal facility at Crown Bioscience following the approved protocols by the Institutional Animal Care and Use Committee. Briefly, xenografted tumors from model CR1197 were implanted subcutaneously on the flanks of female BALB/c mice aging 6–7 weeks. Drug treatment started when tumors reached 100–300 mm^3^, and TVs were measured every 3 or 4 days for 4 to 7 weeks or until they reached 2,000 mm^3^, at which point mice were euthanized. Both single-drug treatments were given twice weekly via intraperitoneal injection at 15 mg/kg (15 mg per 1 kg mouse body weight) for cetuximab and 10 mg/kg for palbociclib. The combo treatment used the same dosing schedule and dosages of the two drugs. Data from all other *in vivo* combination studies were from cited papers with study details therein (4, 35).

### Software and Data Availability

An R package named invivoSyn was developed for the synergy evaluation of the 4-arm *in vivo* combination study. It can calculate CI and SS for several methods including ones based on eGR, TGI, and line mixed models. invivoSyn was written in R and can be easily integrated with other bioinformatics tools. The package is freely available for downloading at https://github.com/maobinchen/invivoSyn. All data and R code for figures are available at https://github.com/maobinchen/invivoSyn_manuscript.

## Results

### Overview of Approach

Our method for evaluating *in vivo* synergy, named invivoSyn, is based on an unbiased efficacy metric called *eGR* that measures the average tumor GR in the entire study duration (Fig. 1A), as detailed in Materials and Methods. It makes no assumptions on tumor growth behaviors and requires no curve fitting, and can be directly calculated from data of TVs and measuring days. A standard *in vivo* combination study has four treatment groups (C, A, B, or A+B), each drug treatment (A, B, or A+B) has a fixed dose (Fig. 1B). A group generally consists of multiple mice with variations in starting TVs and growth behaviors; an average exponential growth curve, with the mean eGR as the rate constant, is drawn regardless of the shapes of actual individual growth curves in light of the interpretation of eGR (Fig. 1C). TV is then used as the measurement for surviving cells to calculate relative survivals (i.e., surviving fractions) in groups A, B, and A+B with respect to group C. Subsequently, the CI and SS are calculated under a synergy model, along with *P* values and 95% confidence intervals (Fig. 1D).

### Validating *In Vitro* Drug Synergy in *In Vivo* Combination Studies

Jaaks and colleagues performed a large-scale drug combination screen in cancer cell lines and identified a set of drug pairs with synergy in certain cell lines (4). SN-38, the *in vivo* active metabolite of TOP1 inhibitor Irinotecan, showed strong synergy with the CHEK1 inhibitor rabusertib under the Bliss model in multiple colon cell lines. The authors subsequently performed 4-arm *in vivo* combination studies in NOD/SCID mice engrafted with three colon cell lines (SW837, SNU-81, and LS-1034) for irinotecan and rabusertib, and observed that tumors shrunk more under the irinotecan + rabusertib combo treatment than the irinotecan treatment. However, such *in vivo* validation studies only showed that synergy exists under the HSA model but not the more stringent Bliss model. HSA synergy exists merely if the combo treatment is better than both single-drug treatments, while Bliss synergy requires that the effects of two drugs are more than additive, that is, the surviving cell percentage under the combo treatment is smaller than the multiplicative product of the surviving cell percentages under the two single-drug treatments.

We now show that our method invivoSyn is able to detect Bliss synergy in the three xenograft studies, with an emphasis on the TV data structure. We also compared our results with those from CombPDX, a recently developed method that calculates CI at individual days (so called local CI) when all four groups have TV data. The average of local CI values is the global CI. We note that both invivoSyn and CombPDX confirmed HSA synergy in all three studies.

**FIGURE 2:**
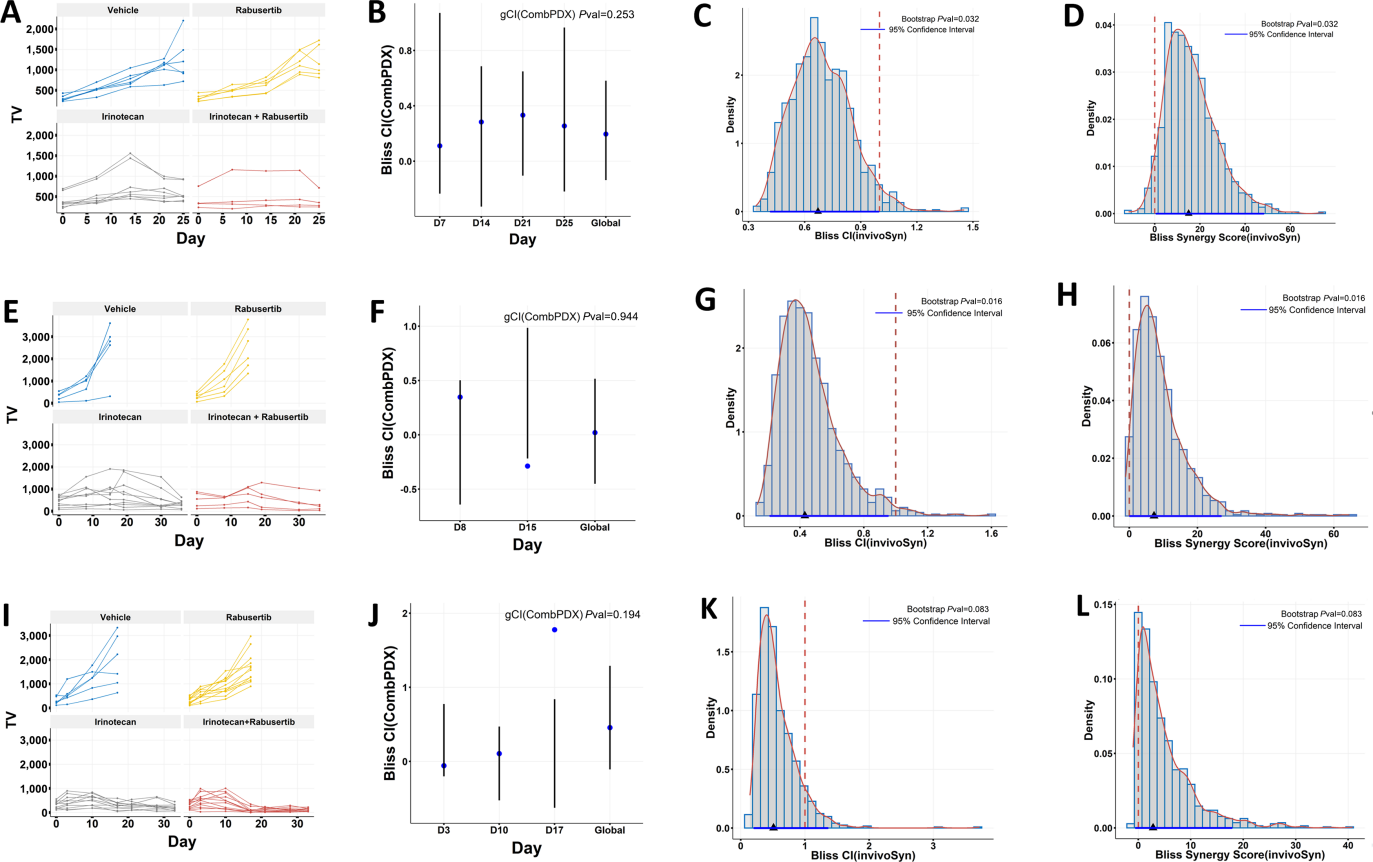
Validating *in vitro* drug synergy in *in vivo* combination studies. The TOP1 inhibitor irinotecan and the CHEK1 inhibitor rabusertib showed strong synergy in three colon cell lines, and were further tested in corresponding cell line–derived xenografts (4). The *in vivo* combination effects were reanalyzed by the CombPDX and invivoSyn methods for the three xenografts: SW837(**A–D**); SNU-81 (**E–H**); LS-1034 (**I–L**). For each xenograft, four graphs from left to right sequentially show tumor growth curves (**A**, **E**, **I**); local CI and gCI values with 95% confidence intervals and *P* values under the Bliss independence model by the CombPDX method (**B**, **F**, **J**); density distribution of the Bliss CI values based on 1,000 bootstrap resamples, along with the actual CI value (black triangle) as well as its 95% confidence interval (blue line) and *P* value, all by our invivoSyn method (**C**, **G**, **K**), where a red dashed vertical line indicates additive effect (1 for CI and 0 for SS); similar information for Bliss SSs by the invivoSyn method (**D**, **H**, **L**). Results in **B**, **F**, and **J** were drawn using output from the CombPDX website. Blue dots indicate local CI values, vertical lines show the 95% confidence interval. Some local CI values are erroneously not in the corresponding 95% confidence intervals.

The SW837 xenograft study had full TV data for all mice in all days until the study was terminated at day 25 (Fig. 2A). This study has an unbalanced design with different numbers of mice in the four groups, with large variations of starting TVs in the irinotecan and combo groups. CombPDX reported no Bliss synergy either at any day or globally (*P* = 0.253 for global CI; Fig. 2B), which indicates a lack of statistical power and high fluctuation for single day–based synergy methods. In contrast, invivoSyn detected strong Bliss synergy (*P* = 0.032; Fig. 2C and D). The CI is 0.672, which means that under the combo treatment, the observed TV is only 67.2% of the expected TV when there is only additive effect. The SS is 15.0, meaning that TV is reduced an additional 15% under the combo treatment due to drug synergy. All calculations used TV data from day 21.

The SNU-81 xenograft study exhibited a more typical tumor growth kinetics in mouse studies (Fig. 2E). Tumor grew fast in the vehicle group and the rabusertib group such that TV data were only available in the first 15 days after dosing started, while the irinotecan and the combo groups had TV data available for 36 days. The uneven TV data structure posed a challenge for CombPDX, which could not use TV data beyond day 15 and failed to account for the long-lasting effect of the combo treatment (Fig. 2F, *P* = 0.944). In contrast, invivoSyn was able to detect significant Bliss synergy using all available TV data with *P* value 0.016 (Fig. 2G and H), demonstrating its robustness and flexibility in handling uneven tumor growth data.

The LS-1034 xenograft study had a similar TV structure as the SNU-81 study (Fig. 2I). However, irinotecan treatment was more effective to LS-1034 such that the efficacy difference between the irinotecan and the combo treatments was not pronounced and evaded detection by CombPDX (Fig. 2J). invivoSyn was able to identify Bliss synergy with borderline significance (*P* = 0.083; Fig. 2K and L).

### Detecting Drug Combination Effects Directly from *In Vivo* Studies

**FIGURE 3:**
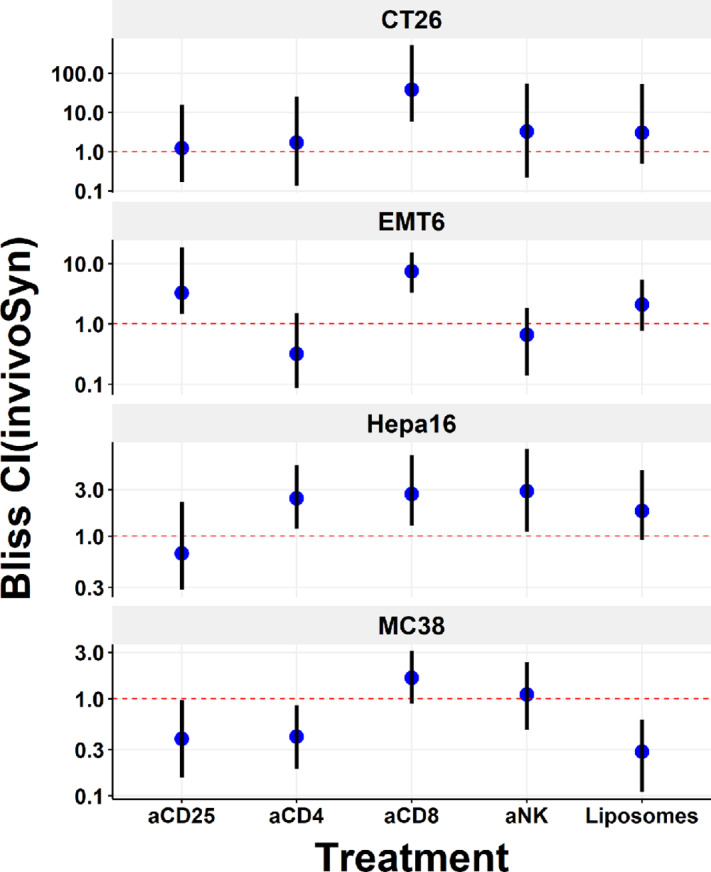
Determining drug combination effects directly from *in vivo* studies. Combination effects were determined for an anti-mouse PD-1 antibody and five therapeutic agents that deplete tumor-infiltrating leukocytes in four syngeneic models (cancer types: CT26 colon, EMT6 breast, Hepa16 liver, MC38 colon). aCD25: antibody for CD25^+^ cells enriched for Treg; aCD4: antibody for CD4^+^ T cells; aCD8: antibody for CD8^+^ T cells; aNK: antibody for removing NK cells; Liposomes: clodronate liposomes for depleting TAM. CI values (blue dots) and 95% confidence intervals (black lines) were estimated under the Bliss independence model by the invivoSyn method, the red dashed line (CI = 1) indicates additive effect, CI > 1 is antagonistic effect, CI < 1 is synergistic effect.

We previously performed a series of *in vivo* combination studies using an anti-mouse PD-1 antibody and one of five other antibodies/agents that deplete CD8^+^ T cells, CD4^+^ T cells, regulatory T (Treg) cells, natural killer (NK) cells, and tumor-associated macrophages (TAM), respectively, in four anti-PD-1 antibody-responsive syngeneic models to show that they have different mechanisms of actions under the anti-PD-1 treatment (35). Such tumor-infiltrating leukocyte (TIL) depletion experiments cannot be performed in cell lines, and we applied our method to directly assess combination effects between the anti-PD-1 antibody and the other agents (Fig. 3).

Strong antagonism was common in all four syngeneic models under the anti-PD-1 and anti-CD8 combo treatment, which indicates CD8^+^ cytotoxic TILs play a major role in the anti-PD-1 antibody's therapeutic efficacy. The models then exhibited distinctive response patterns under the other four combo treatments. Notably, the depletion of CD4^+^ T cells, Treg cells, and TAM significantly synergizes with the anti-PD-1 antibody in MC38; the depletion of CD4^+^ T cells, NK cells, and TAM all antagonizes with the anti-PD-1 antibody in Hepa1–6.

In the previous report, we only compared treatment effects between the anti-PD-1 treatment and a combo treatment to show that one was superior in suppressing tumor growth (35). The quantitative invivoSyn analysis unambiguously classified combination effects into additivity, synergism, and antagonism that gave us a better understanding of how intrinsic tumor immunity affects immunotherapies in different syngeneic models, thereby, facilitating the development of distinctive strategies in using them to design effective combination immunotherapies.

### InvivoSyn Detects Synergy with High Statistical Power and Low FDR

We demonstrate the superior performance of invivoSyn in detecting synergy under different scenarios using simulated data. The results also serve as guidance for designing effective *in vivo* studies by considering statistical power, FDR, and sample size.

#### InvivoSyn has High Power and Low FDR for Regular Tumor Growth Patterns

**FIGURE 4:**
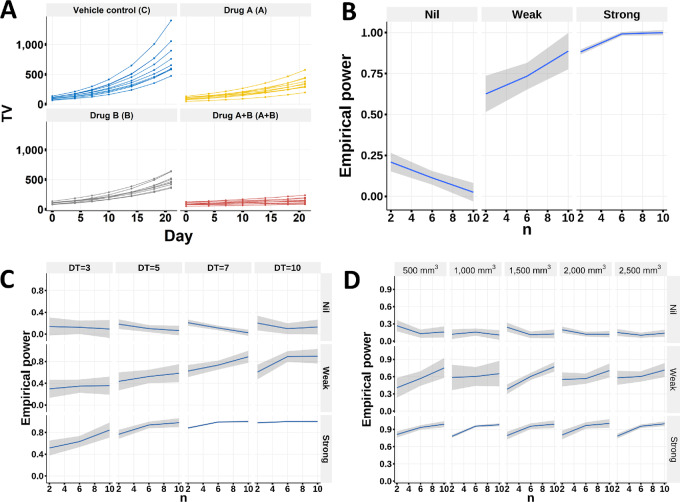
invivoSyn detects synergy with high statistical power and low FDR under regular tumor growth patterns. **A** and **B,** Simulations were conducted to obtain empirical statistical power to detect Bliss synergy with respect to the number of mice n in a group, assuming equal number of mice in all four groups, under regular tumor growth where tumors in a group either grow or shrink with variations in GR. TV is measured twice a week for 3 weeks (on days 0, 4, 7, 10, 14, 18, 21) assuming that tumor DT is 7 days, and the TV cutoff is set to 3,000 mm^3^. **C,** Simulation results at four DT values (DT = 3, 5, 7, 10 days) with all other simulation parameters same as in A, showing that power increases with respect to DT for a given n value for both weak and strong synergies, while for nil synergy, the power is always low. **D,** Simulation results under five tumor volume cutoffs (500, 1,000, 1,500, 2,000, 2,500 mm^3^) for mouse euthanasia with DT = 5 days, showing that power is not strongly affected by TV cutoff.

In most *in vivo* combination studies, tumors exhibit regular growth patterns in the sense that all tumors in a treatment group either grow or shrink with variations in GR (Fig. 4A). We conducted a statistical power analysis using three simulated datasets with strong, weak, and nil synergy under the Bliss independence model with a 7-day tumor doubling time (DT) and a cutoff of 3,000 mm^3^ for mouse euthanasia (Fig. 4B). Under the nil synergy setting, the power is low so that we will not erroneously claim synergy (i.e., low FDR). Under the weak synergy setting, the power increases steadily with sample size, and a high power can be achieved with modest sample sizes (e.g., 8–10 mice per group). Under the strong synergy setting, the power is larger than 80% even with a small sample size (2 mice per group), and becomes complete when there are 6 mice per group, so synergy can be easily detected.

The statistical power is also affected by tumor GR and cutoff of TV for mouse euthanasia. Tumor GR is represented by tumor DT in days. We performed simulation for DT = 3, 5, 7, 10 days, and observed that power increases with respect to DT for a given number of mice n (Fig. 4C). Other things being equal, a fast-growing tumor would need more mice to achieve a comparable power than a slow-growing tumor. This is likely due to the larger variation of TV between mice for fast-growing tumors; therefore TV based metrics, such as eGR, also have larger variation. We also performed simulation with varied TV cutoffs for mouse euthanasia. The results show that power is not strongly affected by the cutoff (Fig. 4D). This is expected as long as there are enough datapoints (i.e., number of measurement days) to estimate eGR and the TV cutoff is not too small (e.g., only slightly larger than the initial TV).

#### InvivoSyn has High Power for Delayed Treatment Effect

**FIGURE 5:**
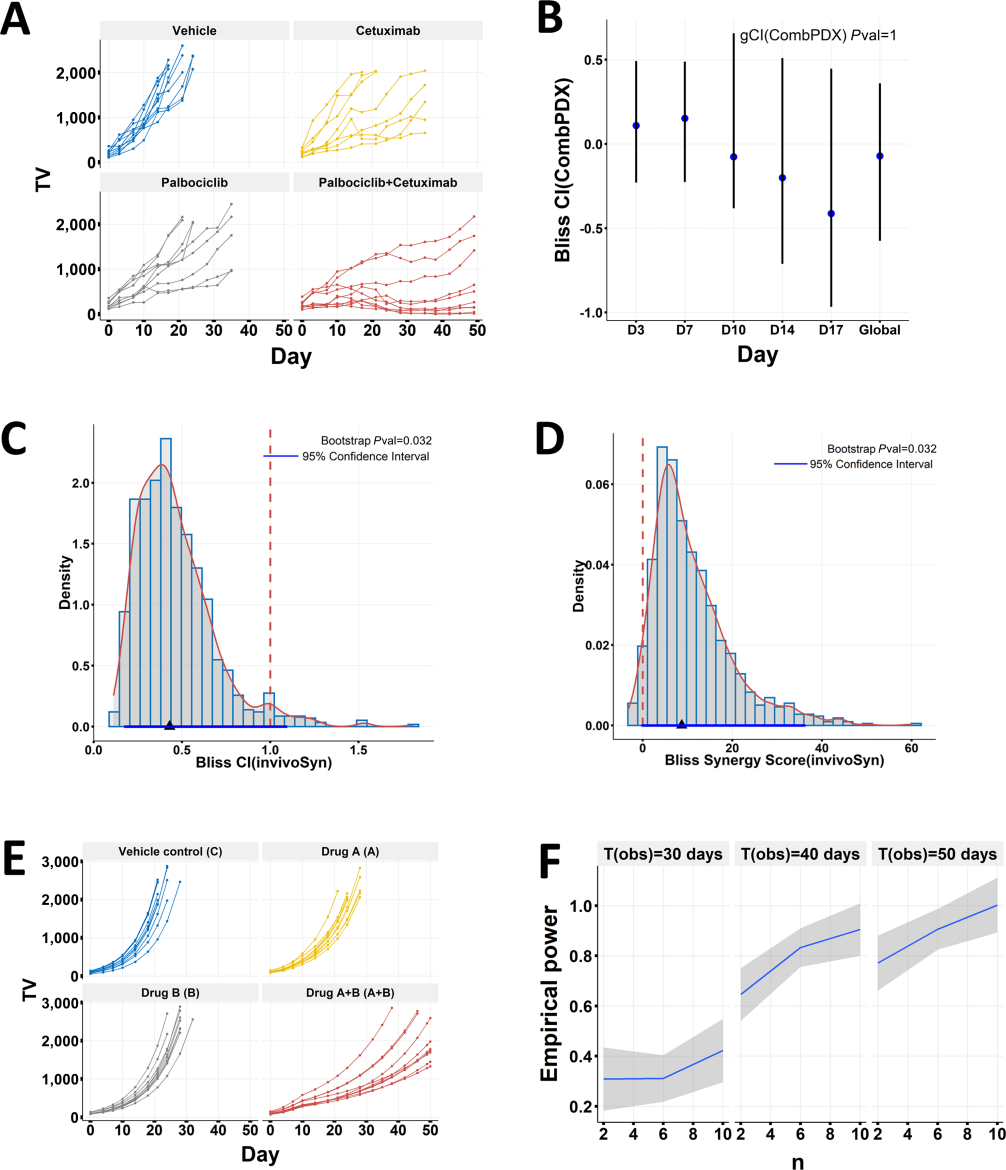
Assessing synergy with delayed treatment effect. **A,** A 4-group *in vivo* combination study of the CDK4/6 inhibitor palbociclib and the EGFR inhibitor cetuximab in a colon PDX model CR1197, delayed treatment effect was observed in the combo treatment group. There are 10, 8, 9, 10 mice in the vehicle, cetuximab, palbociclib, and the combo groups, respectively. **B,** No synergy under the Bliss independence model was reported by the CombPDX method. **C** and **D,** Strong synergistic effect under the Bliss independence model was detected by the invivoSyn method, as evidenced by both the CI and SS. **E** and **F,** Simulations were conducted to obtain empirical statistical power to detect Bliss synergy with respect to the number of mice n in a group, assuming equal number of mice in all four groups, but with delayed treatment effect in the A+B combo group such that tumors exhibit reduced GR after day 10 at which drug effect takes place. TV is measured twice a week for 3 weeks (on days 0, 4, 7, 10, 14, 18, 21) assuming that tumor DT is 7 days, and the TV cutoff is set to 3,000 mm^3^. The simulation results show that sufficient power (>0.8) is only achieved with long study durations [i.e., observation days or T_obs_ = 40 and 50 days]. In C and D, the histogram, overlaid by a red fitted density curve, shows the distribution of 1,000 bootstrap values for CI or SS; the red dashed vertical line indicates additive effect (1 for CI and 0 for SS); the black triangle marks the calculated value for CI or SS; the blue horizontal line indicates the 95% confidence interval. invivoSyn is the name of our method as well as the software package implementing it.

The delayed treatment effect is a phenomenon where drug effect takes place sometime after administration, which may be caused by the absorption, distribution, metabolism of the drug or its mechanism of action. We illustrate this by an example. The combination of the CDK4/6 inhibitor palbociclib and the EGFR inhibitor cetuximab was proposed as a potential strategy to overcome cetuximab resistance by targeting cyclin D1 deregulation (28). To evaluate their combination effect, we conducted a study using a colon PDX model CR1197 (Fig. 5A). We noticed that some mice in the combo group exhibited a delayed response that resulted in tumor regression then regrowth at the late stage of the study, whereas the vehicle group had a shorter study duration (49 vs. 24 days). CombPDX detected no trace of Bliss synergy (*P* value = 1; Fig. 5B), due to its incapability of capturing the late-stage effect in the combo treatment. *invivoSyn* identified a significant Bliss synergy with *P* value 0.032 (Fig. 5C and D).

We again performed power analysis by simulating three datasets with strong, weak, and nil synergy under the Bliss model, assuming that drug effects take place 10 days after administration for treatment A and the combo treatment (Fig. 5E). The power increases steadily by both sample size and study duration, but is insufficient if the study is terminated too early at day 30. When the study duration is extended to 40–50 days, power is sufficiently large (>0.8) with fairly smaller sample size (Fig. 5F). These results suggest that invivoSyn has adequate power to identify the long-term synergistic effect, provided that the follow-up period is sufficiently long after the drug effect kicks in.

#### InvivoSyn has Low FDR for Tumor Regrowth Under Combo Treatment

**FIGURE 6:**
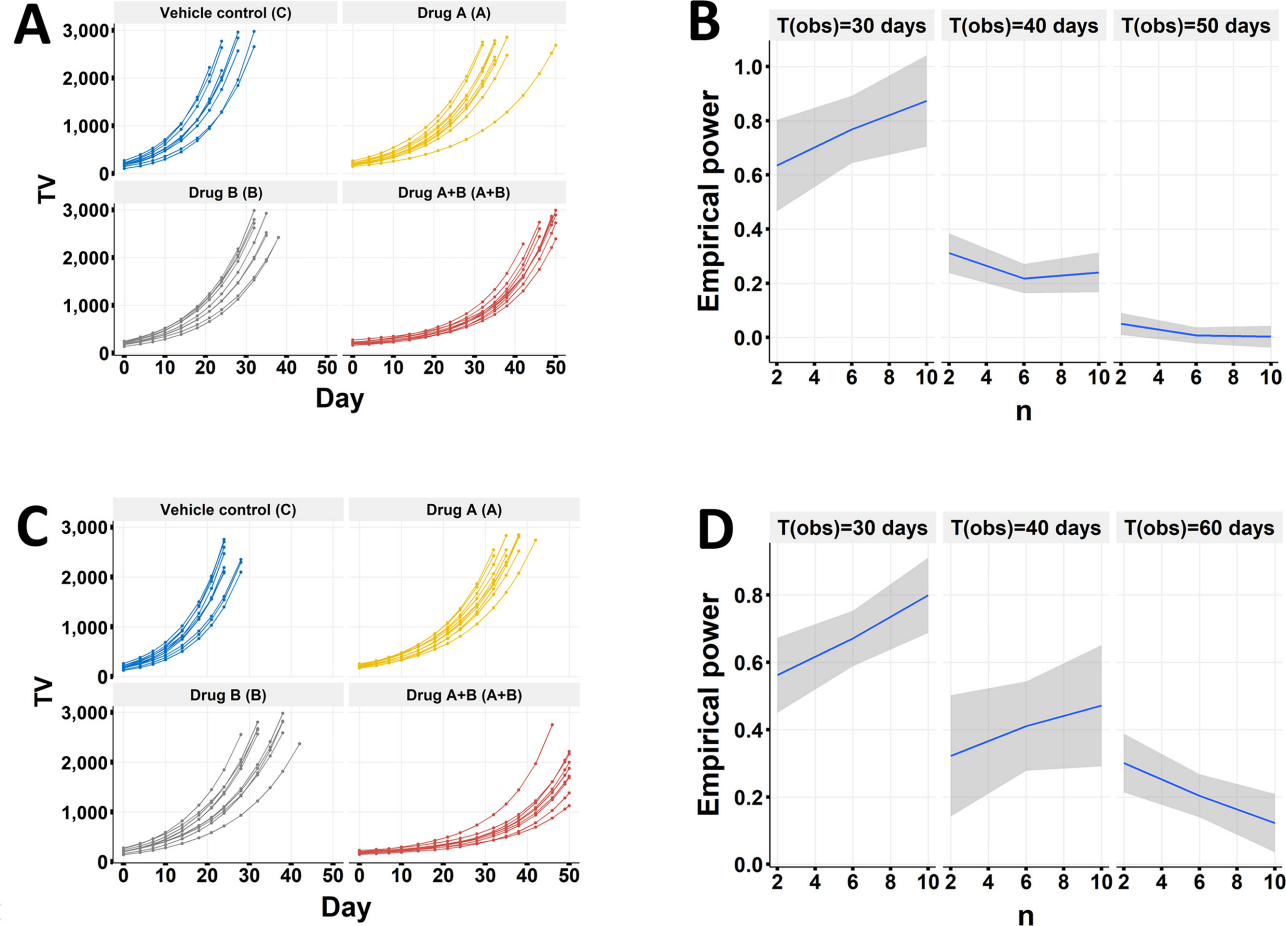
Assessing synergy with tumor regrowth. Simulations were conducted to obtain empirical statistical power to detect Bliss synergy with respect to the number of mice n in a group, assuming equal number of mice in all four groups but with tumor regrowth. TV is measured twice a week for 3 weeks (on days 0, 4, 7, 10, 14, 18, 21) assuming that tumor DT is 7 days, and the TV cutoff is set to 3,000 mm^3^. No synergy is imposed. **A** and **B,** Tumors regrow under combo treatment due to intrinsic resistance where 10% of tumor cells are intrinsically resistant to treatments A and A+B. **C** and **D,** Tumors regrow under combo treatment due to induced resistance where 10% of tumor cells resume growth with initial GR at day 10 under treatments A and A+B. In both scenarios, empirical power exhibits dependence on both mouse number n and observation time T_obs_. When T_obs_ is sufficiently long (40–60 days), invivoSyn shows low empirical power, therefore low FDR, for synergy detection.

Tumor regrowth occurs when a small portion of tumor cells possess intrinsic or induced resistance, and manifests as V-shape growth curves. We simulated intrinsic resistance by assuming that 10% of tumor cells are not affected in all three drug treatment groups (A, B, and A+B; Fig. 6 and B), and simulated the induced resistance by assuming 10% of tumor cells resume growth at day 10 after drug administration under treatments A and A+B (Fig. 6C and D). No synergy was imposed for the combo treatment in the simulations, so there is only additive effect. Because no synergy exists, invivoSyn should have fairly low FDR, which in this case equals low statistical power. We observed that in both simulations, statistical power, hence FDR, is high if the study duration is only 30 days because by then the effect of tumor regrowth is still not strong enough, but when the duration extends to 40–50 days, power and FDR decrease steadily, and quickly approach 0 when sample size goes to 10 and study duration is 60 days (Fig. 6B and D). Overall, our results suggest that invivoSyn has low FDR for detecting synergy even if tumors regrow, as long as the combination study is well designed with enough mice and sufficiently long study duration.

### Synergy Metrics are Both Time and Dose Dependent Under the Bliss Independence Model

Drug synergy, either *in vivo* or *in vitro*, can be best characterized using the full factorial or matrix design with longitudinal measurement of cell/tumor proliferation. The MuSyC framework unifies a variety of synergy metrics and separates potency synergy and efficacy synergy such that the two synergies are objective metrics purely determined by the interactions of two drugs, and are neither dose nor time dependent (13). However, in practice, nearly all *in vivo* combination studies use the 4-group design for its simplicity and relatively low cost. Subsequently, the *in vivo* synergy can only be analyzed by a limited number of synergy models, most notably the Bliss independence model. We now show that under the Bliss independence model, CI is both time and dose dependent by comparing the 4-group *in vivo* combination study with the fixed-ratio *in vitro* combination study. Thus, Bliss synergy, including CI and SS, should be used and interpreted with caution. All synergies thereafter in this section refer to Bliss synergy.

**FIGURE 7:**
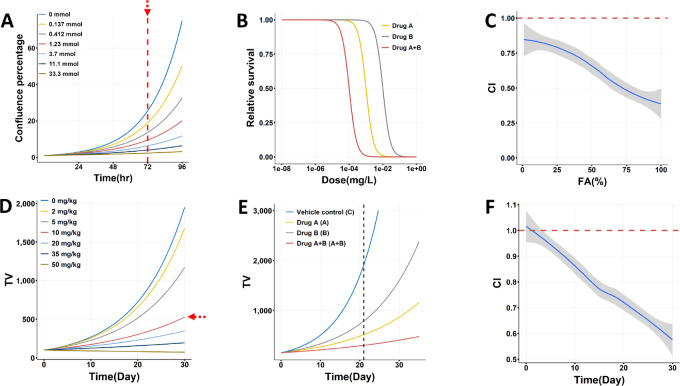
A unified view of *in vitro* and *in vivo* synergy. Drug efficacy measurement, study design, and CI calculation are illustrated for the typical fixed-ratio *in vitro* combination study (**A–C**) and 4-group *in vivo* combination study (**D–F**). A, In *in vitro* cell assays, drug efficacy is measured at a single timepoint (e.g., 72 hours, the red dashed line) for a series of drug concentrations, where higher concentrations exert stronger antiproliferative effect as quantified by confluence percentage. B, In an *in vitro* combination study with the fixed-ratio design, dose–response curves are inferred for drugs A, B, A+B, using efficacy measured at 72 hours. C, The CI is estimated for a range of drug concentrations where high concentration causes higher fraction of affected (FA) cells. Therefore, the CI is dose dependent. D, In *in vivo* studies, drug efficacy is measured at a fixed dose (growth curve pointed by the red arrow) for the study duration. E, In an *in vivo* combination study with the 4-group design, tumor growth curves were obtained for vehicle control, drugs A, B, and A+B, each with a fixed dose. CI can be calculated at a particular day (cf. Eq. 4). F, The CI is calculated for a range of days in the study duration. Therefore, the CI is time dependent. We note that exponential growth kinetics is assumed for both cell proliferation (A) and tumor growth (D) under drug treatment, and well before reaching maximal values constrained by nutrient and space. Previous studies have provided theoretical (equation 5 in ref. 33) and empirical justifications (31).

At first sight, *in vitro* and *in vivo* combination studies are very different, the former measure cell density in a plate well under a drug treatment with multiple concentrations to infer the dose–response curve, the latter measure tumor volume in a mouse under a drug treatment with a fixed dose for a duration of weeks to months to obtain the tumor growth curve. However, the two types of studies can be unified on the basis of growth inhibition (Fig. 7). Specifically, we assume exponential growth kinetics for both cell proliferation (Fig. 7A) and tumor growth (Fig. 7D) under drug treatment and well before reaching maximal values constrained by nutrient and space. Previous studies have provided theoretical (equation 5 in ref. 33) and empirical justifications (31). Subsequently, cell/tumor GR under the exponential growth kinetics is used as a drug efficacy metric, and CI, when defined here via relative survival of tumor cells, is both dose and time dependent, as shown below.

In an *in vitro* combination study, drug efficacy is measured at a particular timepoint (e.g., 72 hours) for a series of drug concentrations (Fig. 7A, as pointed by the vertical red arrow). Cells proliferate more slowly at higher drug concentrations due to stronger inhibition, as evidenced by the growth curves. The dose–response curves are inferred for drugs A, B, A+B, commonly by a 4-parameter logistic function (Fig. 7B), then the CI is estimated for a range of drug concentrations (Fig. 7C). It is a one-timepoint multi-dose study.

In an *in vivo* combination study, drug efficacy is measured at a fixed dose level for the study duration (Fig. 7D, as pointed by the horizontal red arrow). Tumors grow slower under a more potent drug treatment. The four tumor growth curves are drawn for vehicle control, drugs A, B, and A+B, by connecting adjacent tumor volume datapoints (Fig. 7E), then the CI is estimated at individual days of the study duration (Fig. 7F). At different days, the values of CI and SS vary, but the *P* value is unchanged (It is therefore more meaningful to use *P* value to assess synergy.). It is a one-dose multi-timepoint study.

It is evident that *in vitro* and *in vivo* studies are assessing combination effects from different angles so that either only describe part of the synergy landscape. The *in vitro* studies show that synergy is dose dependent; the *in vivo* studies show that synergy is time dependent, which is explicit in the CI definitions that contain the time variable (Eqs. 4 and 5). Jointly, we know that the CI, as well as the SS, is both time and dose dependent. If we choose a different day (e.g., 96 hours instead of 72 hours in Fig. 7A) for an *in vitro* study, we will get a different CI value at a given dose level in Fig. 7C. Similarly, if we use different drug doses in an *in vivo* study, we will get a changed CI value at a given day in Fig. 7F.

The CI is defined in essentially the same way for both *in vitro* and *in vivo* combination studies due to the parallelism shown in Fig. 7. Specifically, *in vitro* drug efficacy is quantified by the relative survival of drug-treated cells using untreated cells as the reference, and *in vivo* drug efficacy is quantified by relative tumor volume, as a proxy for relative survival of tumor cells, of drug-treated tumors using vehicle-treated tumors as the reference (Eqs. 1–3). Nonetheless, if two drugs have strong *in vitro* synergy, significant *in vivo* synergy can be observed with proper study designs, as we explain more in Discussion.

## Discussion

Methodologies for evaluating drug combination effects have been well developed for *in vitro* assays, but are still under active research for *in vivo* animal studies. This study establishes a well-founded method to investigate *in vivo* combination studies and provides a unified view of *in vitro* and *in vivo* synergy under the Bliss independence model.

Existing methods for evaluating *in vivo* combination effects fall into two categories, ones using differential and kinetic equations to model tumor growth, and ones directly comparing TV at a single day. As we explained previously and showed in cases studies, both approaches have fundamental drawbacks that limit their ability to detect drug synergy accurately and sensitively. Our method demonstrated superior performance in both real-world studies and simulated datasets, and it imposes no constraint on tumor growth kinetics and uses TV data from all days for all mice. Our method is based on eGR, an unbiased metric of *in vivo* antiproliferative drug effect (30), which is similar to two unbiased metrics of *in vitro* antiproliferative drug effect, the DIP rate proposed by Harris and colleagues (33), and the drug-induced GR inhibition by Hafner and colleagues (32), and it offers an easy algebraic way for calculating GR for *in vivo* studies.

Assessing *in vivo* synergy had been largely treated as a separate effort from assessing *in vitro* synergy, and terminologies, such as CI, were often defined with intrinsically different interpretations. In this work, we showed that a parallelism exists between *in vitro* and *in vivo* combination studies such that not only terminologies can be defined in conceptually the same way, but also synergy models can be applied equally (Fig. 7). We further emphasize that synergy, when represented by CI, is both time and dose dependent; a typical fixed-ratio *in vitro* combination study evaluates the combination effect at a single timepoint, while a typical 4-group *in vivo* combination study evaluates the combination effect at single-dose levels.

Because of the time-vs-dose difference between typical *in vitro* and *in vivo* combination studies, cautions must be exercised in applying methods initially developed for *in vitro* studies to *in vivo* studies. For example, when the Chou-Talalay method is applied to *in vivo* studies (9, 36), it requires multiple dose levels for each drug, so there will be may groups (for an example, see ref. 37), while in practice, we normally have only four groups, not only to save cost but to focus on the selected drug dose levels.

There are a number of synergy models and methods for evaluating synergy, only some are applicable to the typical 4-group in *vivo* combination studies. Models requiring multiple doses are not applicable, such as the Loewe additivity model and the response surface-based methods (18, 38). In our opinion, the HSA and the Bliss models are best suited for assessing oncology drug combinations under the 4-group design. HSA model is not based on probability theory but more a convenient way to evaluate whether a combo treatment is better than either single treatment, and is, therefore, somewhat abusing the synergy terminology. Nonetheless, a significant HSA synergy means a combo treatment is better than either single-agent treatment, which is often sufficient in the clinical treatment of patients with cancer. A significant Bliss synergy is even stronger and more desired. However, its clinical validation is generally not possible to conduct due to ethical regulations and cost. Therefore, *in vitro* and *in vivo* combination studies are of great importance to detect and confirm Bliss synergy.

There are several recommendations for conducting *in vivo* combination studies. The first one is to select proper drug doses so the tumor inhibition effect is not overly strong. As a numerical example, if the relative survival is 10% and 50% under drug A and drug B treatments, respectively, and there is only additive effect, the relative survival under drug A+B treatment is only 5%. It is therefore hard to detect synergistic effect under which the relative survival is between 0% and 5%, given the measurement error and tumor volume variance. A real-world example is the combination study of irinotecan+rabusertib on the LS-1034 xenograft (Fig. 2I–L). Irinotecan has very strong efficacy by itself, so although the combo treatment is more potent, the *P* value for Bliss synergy is only 0.083, even though the two drugs showed stronger *in vitro* efficacy in LS-1034 than in SNU-81 and SW837. The second one is to use enough but not too many mice and properly long study durations. The power curves in Figs. 4–6 provide general guidance for determining sample size and study duration under various scenarios. The third one is to use combination indices and SSs cautiously, especially when comparing between different xenograft models or comparing drug combinations in the same xenograft model, because these two synergy measures depend on tumor GR and time. As pointed out in the main text, *P* value for the combination effect can be a better indicator because it does not vary by day at which the CI is calculated.
